# “*Candidatus* Uabimicrobium helgolandensis”—a planctomycetal bacterium with phagocytosis-like prey cell engulfment, surface-dependent motility, and cell division

**DOI:** 10.1128/mbio.02044-24

**Published:** 2024-08-27

**Authors:** Carmen E. Wurzbacher, Jonathan Hammer, Tom Haufschild, Sandra Wiegand, Nicolai Kallscheuer, Christian Jogler

**Affiliations:** 1 Department of Microbial Interactions, Institute of Microbiology, Friedrich Schiller University Jena, Jena, Germany; 2 Department of Microbiology, Radboud University Nijmegen, Nijmegen, Netherlands; 3 Cluster of Excellence Balance of the Microverse, Friedrich Schiller University Jena, Jena, Germany; Max Planck Institute for Marine Microbiology, Bremen, Germany

**Keywords:** evolutionary biology, endocytosis, planctomycetes

## Abstract

The unique cell biology presented by members of the phylum *Planctomycetota* has puzzled researchers ever since their discovery. Initially thought to have eukaryotic-like features, their traits are now recognized as exceptional but distinctly bacterial. However, recently discovered strains again added novel and stunning aspects to the planctomycetal cell biology—shapeshifting by members of the “*Saltatorellus*” clade to an extent that is unprecedented in any other bacterial phylum, and phagocytosis-like cell engulfment in the bacterium “*Candidatus* Uabimicrobium amorphum.” These recent additions to the phylum *Planctomycetota* indicate hitherto unexplored members with unique cell biology, which we aimed to make accessible for further investigations. Targeting bacteria with features like “*Ca.* U. amorphum”, we first studied both the morphology and behavior of this microorganism in more detail. While similar to eukaryotic amoeboid organisms at first sight, we found “*Ca.* U. amorphum” to be rather distinct in many regards. Presenting a detailed description of “*Ca.* U. amorphum,” we furthermore found this organism to divide in a fashion that has never been described in any other organism. Employing the obtained knowledge, we isolated a second “bacterium of prey” from the harbor of Heligoland Island (North Sea, Germany). Our isolate shares key features with “*Ca.* U. amorphum”: phagocytosis-like cell engulfment, surface-dependent motility, and the same novel mode of cell division. Being related to “*Ca.* U. amorphum” within genus thresholds, we propose the name “*Ca.* Uabimicrobium helgolandensis” for this strain.

**IMPORTANCE:**

“*Candidatus* Uabimicrobium helgolandensis” HlEnr_7 adds to the explored bacterial biodiversity with its phagocytosis-like uptake of prey bacteria. Enrichment of this strain indicates that there might be “impossible” microbes out there, missed by metagenomic analyses. Such organisms have the potential to challenge our understanding of nature. For example, the origin of eukaryotes remains enigmatic, with a contentious debate surrounding both the mitochondrial host entity and the moment of uptake. Currently, favored models involve a proteobacterium as the mitochondrial progenitor and an Asgard archaeon as the fusion partner. Models in which a eukaryotic ancestor engulfed the mitochondrial ancestor via phagocytosis had been largely rejected due to bioenergetic constraints. Thus, the phagocytosis-like abilities of planctomycetal bacteria might influence the debate, demonstrating that prey engulfment is possible in a prokaryotic cellular framework.

## OBSERVATION

The bacterial phylum *Planctomycetota* has intrigued microbiologists for years, with some suggesting that these bacteria represent a “missing link” between prokaryotic and eukaryotic cells (1–3). Initially, this theory was supported for example by a protein uptake mechanism in *Gemmata obscuriglobus*, resembling to some extent eukaryotic endocytosis (4). However, extensive research over the past decade has largely doubted these hypotheses (5–8). Even though planctomycetes still present an unconventional cell biology, they seemed to generally align with a Gram-negative cell plan (9). However, in 2019, we first described the phylogenetically distinct “*Saltatorellus*” clade (10, 11). A few months later, Shiratori et al., published “*Candidatus* Uab amorphum” (12) later renamed to “*Ca.* Uabimicrobium amorphum” (UA) acknowledging taxonomic rules (13). Showing unprecedented cell biological features, these new strains seem to challenge not only aspects of the planctomycetotal cell plan but also that of diderm bacteria in general. Especially UA’s unique capability of feeding on other bacteria via phagocytosis-like prey uptake contradicts previous predictions based on theoretical calculations as well as features of the Gram-negative cell plan (14, 15). Although other studies have suggested that the proposed energetic barrier preventing bacteria from developing complex traits such as phagocytosis does not exist, living evidence has not been found until now (16, 17). Thus, the discovery of organisms such as UA and the “*Saltatorellus*” clade is of major importance for several fields from biodiversity to evolution.

**Fig 1:**
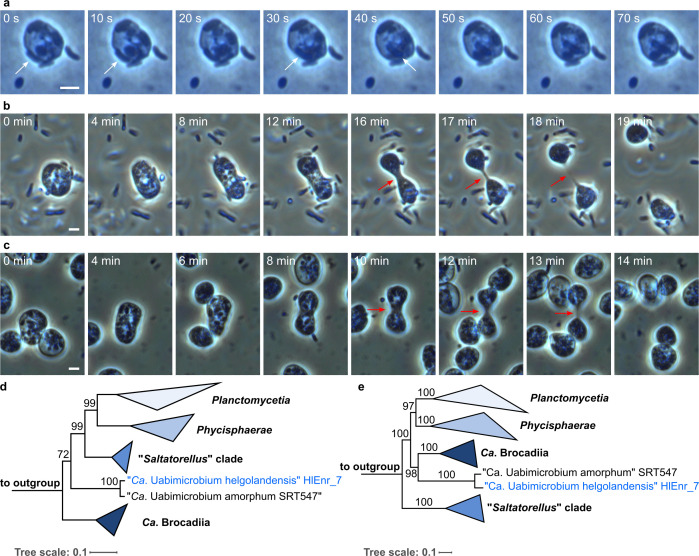
Overview on the cell biology and phylogeny of “*Ca.* U. amorphum” SRT547 as well as the novel isolate “*Ca*. U. helgolandensis” HIEnr_7. Phagocytosis-like uptake of surrounding prey bacteria by “*Ca.* U. amorphum” (large cell) (**a**); white arrows indicate the prey bacterium being internalized. Cell division of “*Ca.* U. amorphum” (**b**), and “*Ca.* U. helgolandensis” HlEnr_7 (**c**). Two opposite cell poles move apart until only a thin, thread-like connection remains (red arrows) that finally disrupts. 16S rRNA gene sequence- (**d**) and multi-locus sequence analysis (MLSA)- (**e**) based phylogenies showing the deep branching of the “*Ca.* Uabimicrobium” clade within the phylum *Planctomycetota*.

Aiming to isolate bacteria possessing similar phagocytosis-like capabilities as “*Ca.* U. amorphum” from the environment, we first needed to assess possibilities for their identification from both morphological and genomic perspectives. Therefore, we obtained a UA culture from the Japan Collection of Microorganisms (JCM 39082). Observing UA under the microscope, its striking resemblance to eukaryotic amoeboid organisms became evident (Fig. 1; Movie S1 and S2). Since this posed a high danger of mistaking amoebae for such bacteria, we screened literature and culture collections for size-wise comparable eukaryotic amoeboid organisms, to study similarities and differences. Based on such morphological criteria, we found *Hartmannella* sp. CCAP 1534/15 and *Squamamoeba japonica* CCAP 1493/1 to be suitable for the comparison.

Despite certain similarities, we noticed clear differences: both, *Hartmannella* sp. and *S. japonica* have a rather uniform cell size (about 20 µm and 6 µm, respectively) and intracellular granulation. In contrast, UA cells differ significantly in size (4–20 µm) and granularity. Furthermore, the locomotion of UA contrasts that of the tested amoebae: while amoebae form pseudopodia to crawl along surfaces, UA cells show no such arm-like protrusions and maintain a mostly round cell shape during crawling. Additional distinctions can be found in their cellular division process: UA cells require a surface to generate the force needed for dividing into two daughter cells (Fig. 1b; Fig. S1; Movie S2). Opposite cell poles move apart until they are only connected by a thin, thread-like structure. Its length is up to three times the cell diameter prior to division initiation and both cell poles continue crawling apart until the structure disrupts. In comparison, division of the two amoebae seemed not to require pulling by the emerging daughter cells, and neither of the tested amoebae formed such long tubules (Fig. S1; Movie S3). Despite the process of prey engulfment itself appearing quite similar (Fig. S2; Movie S1 and S4), further differences can be found in the organisms’ feeding behavior: while UA seemed to engulf every bacterium encountered on the surface, both amoebae frequently let some bacteria escape. Additionally, the amoebae tend to move around a lot, frequently crossing areas where they already fed on bacteria before. In contrast, UA cells move less and instead internalize every bacterium in their proximity. This leads to the formation of “feeding circles”—accumulations of UA cells grazing in a circular line around areas with no prey bacteria left (Fig. S3). While both amoebae seem to sense accumulations of prey and move toward them over long distances, UA cells just feed in their proximity and ignore more distant prey accumulations.

Although morphological distinctions seemed to suffice for identifying further bacteria of prey, we wondered, whether an amoeba contamination could unquestionably be excluded by our sequencing approaches. Therefore, we sequenced the UA cell culture including *Alteromonas macleodii* prey bacteria, a UA DNA sample (JCM), and the two amoebae cultures. For UA samples, only the two expected bacterial genomes were found [NCBI accession number JAZFBE000000000 (*A. macleodii*, prey bacterium) and JAZFBD000000000 (UA)]. The amoebae cultures yielded genome fragments of the two amoebae, multiple prey bacteria, and mitochondria. Thus, our sequencing method is suitable to distinguish amoeba and bacteria and we can confirm that Shiratori et al.’s UA culture is that of a planctomycetotal bacterium.

Using the information obtained from studying UA, we repeated the original enrichment strategy and obtained a close relative from water sampled in Heligoland (North Sea, Germany) (18). Both morphology and behavior of the obtained isolate appear very similar to UA (12): the majority of its cells are 4–6 µm in size, it obligately feeds on other bacteria (Supplementary results), and cells divide like UA (Fig. 1c). Sequencing an enrichment culture, we obtained three bacterial bins, among them the genome of *A. macleodii* (added prey bacterium). A second, 9.3 Mb bacterial genome (CP165719) relates to UA within genus thresholds (Table S1 and S2), for which we propose the name “*Candidatus* Uabimicrobium helgolandensis” strain HlEnr_7 (UH). Pangenome analysis revealed 4,319 shared genes while 2,442 and 2,398 genes were unique for UH and UA, respectively (Fig. S4). Both multi-locus sequence analysis- (MLSA) and 16S rRNA gene-based tree reconstructions demonstrate deep phylogenetic branching of both “*Ca.* Uabimicrobium spp.” within the phylum *Planctomycetota* (Fig. 1). However, this branching pattern might be compromised by DNA G+C content differences between 70% (“*Saltatorellus”* clade), 43% (*Ca*. Brocadiales), and 39% (UA) (19). Such differences affect sequence similarity and thus alignment accuracy, as sequences with similar DNA G+C content have fewer mismatches. This can influence phylogenetic tree construction and gene prediction: a KEGG KOfam analysis of cell division and peptidoglycan synthesis genes revealed the lack of most such genes in UA and UH, indicating that “*Ca.* Uabimicrobium spp.” do not employ a canonical bacterial cell division mechanism (Fig. S5).

**Fig 2:**
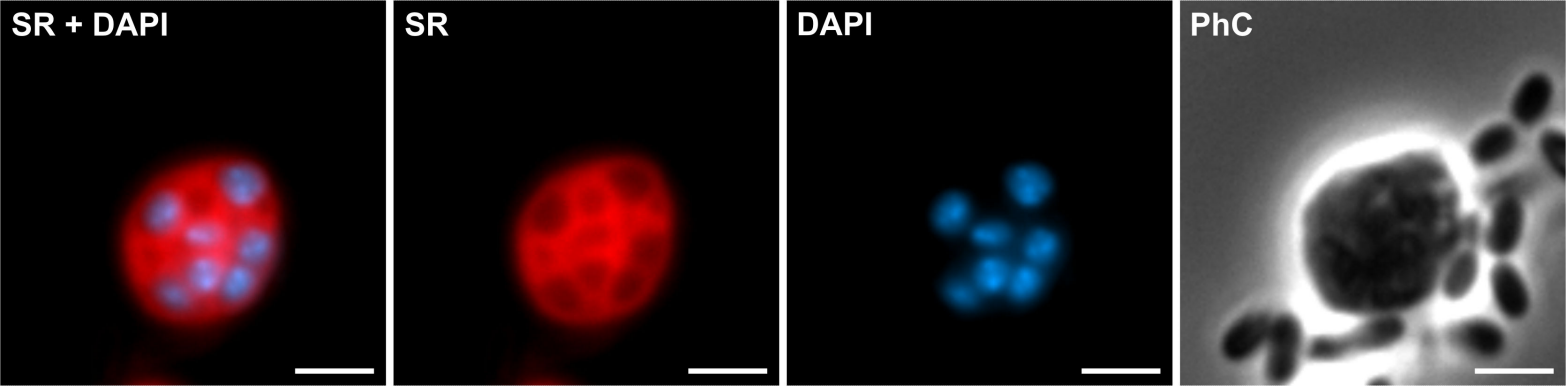
Fluorescent staining of “*Ca*. U. amorphum” with SynaptoRed C2 (SR, membranes) and DAPI (DNA). Prey bacteria do not show fluorescence signals in this figure, since they are in a slightly different focal plane and have much lower fluorescence intensity making them disappear upon brightness and contrast adjustment. Scale bars are 2 µm.

While confirming the findings of Shiratori et al., UH adds further evidence for undiscovered planctomycetotal biodiversity. Besides phagocytosis-like cell engulfment as their hallmark trait, both strains present a novel mode of cell division as well as other unique features that require further investigation. Especially their cell envelope and the process of prey engulfment need careful examination considering the previous critique regarding “true endocytotic invaginations” in Gram-negative bacteria (14). However, membrane coat-like proteins might play a role, as such a protein was recently identified in UA (20). Furthermore, bioenergetics in these cells need to be addressed, since controversial opinions exist on the complexity achievable by prokaryotic cells (15–17). Although further analyses are required, a first stain of membranes and DNA revealed extensive membrane signals inside the cell (Fig. 2), which might serve a similar purpose as mitochondrial cristae increasing the metabolically active surface (15).

Taken together, the study of “*Ca*. Uabimicrobium spp.” indicates the conservation of a phagocytosis-like bacterial uptake mechanism, a predatory lifestyle, and unconventional cell biology. Such traits point toward yet unexplored evolutionary complexities and bioenergetic principles of *Planctomycetota* bacteria.

## Data Availability

The re-sequenced genome of “Ca. U. amorphum” SRT547 is available from NCBI under accession number JAZFBD000000000. The genome of “Ca. U. helgolandensis” HlEnr_7 was deposited under accession number CP165719. The 16S rRNA gene sequences were deposited in GenBank under the accession numbers OR886618 (“Ca. U. amorphum” SRT547) and OR832863 (“Ca. U. helgolandensis” HlEnr_7).

## SUPPLEMENTAL MATERIAL

Supplemental Information mbio.02044-24-s0001.docx Supplemental text, tables, figures, and movie legends.

Movie S1 mbio.02044-24-s0002.avi Endocytosis-like prey uptake of “*Ca*. U. amorphum” (large cells).

Movie S2 mbio.02044-24-s0003.avi Cell division of “*Ca*. U. amorphum.”

Movie S3 mbio.02044-24-s0004.avi Cell division of *Hartmannella* sp. CCAP 1534/15.

Movie S4 mbio.02044-24-s0005.avi Phagocytosis of *Squamamoeba japonica* CCAP 1493/1.

ASM does not own the copyrights to Supplemental Material that may be linked to, or accessed through, an article. The authors have granted ASM a non-exclusive, world-wide license to publish the Supplemental Material files. Please contact the corresponding author directly for reuse.
