# Supplementary material for: “Candidatus Uabimicrobium helgolandensis”—a planctomycetal bacterium with phagocytosis-like prey cell engulfment, surface-dependent motility, and cell division
Source: mBio. 2024 Aug 27;15(10):e02044-24. doi: 10.1128/mbio.02044-24 (PMC11481906; doi:10.1128/mbio.02044-24)
Supplement: Supplemental Information — Supplemental text, tables, figures, and movie legends. [file mbio.02044-24-s0001.docx]

Supplementary Information

Supplementary Material and Methods

Organisms and culture conditions

The co-culture of *Ca.* U. amorphum and *A. macleodii* was retrieved from the Japan Collection of Microorganisms (JCM), where it is available as JCM 39082. Standard cultivation of the co-culture was carried out in JCM Medium 1273 with artificial seawater from JCM Medium 1118 at 24 °C. Both amoebae, *Hartmannella* sp*.* CCAP 1534/15 and *Squamamoeba japonica* CCAP 1593/1, were retrieved from the Culture Collection of Algae and Protozoa (CCAP, Scotland, United Kingdom) and were routinely cultured in Artificial Seawater for Protozoa medium (ASWP, recipe by CCAP) at 20 °C.

**Light Microscopy**

Light microscopic analyses were performed in a Nikon Eclipse Ti2 inverted microscope equipped with a Nikon DS-Ri2 Camera (coloured Phase contrast images) or a Hamamatsu ORCA- Flash4.0LT camera (fluorescence images, black and white Phase contrast images) and a Nikon CFI Plan Apochromat Lambda 100X Ph3 oil immersion objective (numerical aperture 1.45). Brightness and contrast were adjusted by eye using FIJI (Version 2.14.0).

In general, two different methods for sample preparation were employed: For observing locomotion of the cells, the respective cultures were grown in Glass bottom dishes (GBDs, Nunc^TM^), which were then used for microscopy. Secondly, to reduce axial movement of the cells, specimens were immobilised on a medium-supplemented 1% (w/v) agarose cushion. To reduce evaporation, the cover glass was sealed with VLAP (33% (w/w) vaseline (petrolatum), 33% (w/w) lanoline, 33% (w/w) paraffin) against the slide. Timelapse analyses were performed at the same temperatures as standard cultivation.

For staining membranes and DNA, 0.5 μL SynaptoRed^TM^ (stock solution 3 mg/mL) and 1.2 μL 4′,6-diamidino-2-phenylindole (DAPI stock solution 500 μg/mL) were added to a growing culture of 100 µL, which was subsequently incubated for 1 h at 24 °C in darkness. Before imaging, the culture was washed three times with 100 μL of JCM Medium 1273 without LB Broth.

Whole Genome Amplification (WGA)

WGA was performed using the REPLI-g Single Cell Kit from Qiagen in 12mm GBDs (Nunc^TM^). Cells were grown in a GBD until no prey bacteria were attached to the surface anymore. Then, the GBD was flushed four times with JCM Medium 1273 without LB Broth (cell density was observed under the microscope before removing the liquid from the last washing step). Afterwards, cells in the GBD were washed twice with autoclaved ddH_2_O before proceeding with cell lysis. All steps were executed with the volumes for two reactions. For cell lysis, the GBD was then incubated for 10 min each at 4 °C, -20 °C, -80 °C and 65 °C. After addition of the master mix, DNA was amplified for 8 h.

DNA purification and debranching

DNA purification and restriction were performed following Oxford Nanopore’s Ligation sequencing gDNA – whole genome amplification protocol (version: WAL_9154_v112_revF_09Feb2022). As double reaction volumes were used for WGA, purification was performed with double volumes as well. Briefly, DNA was bound to AMPure XP beads (Beckman Coulter), pelleted on a magnet, washed with 70% (v/v) ethanol, and finally resuspended in nuclease-free water. The DNA concentration was determined using the Qubit^TM^ 1x dsDNA BR kit (Thermo Scientific). Subsequently, DNA was debranched as four separate samples using NEB T7 Endonuclease I; incubation at 37 °C was prolonged to 60 min. The following DNA purification deviated slightly from the protocol: Instead of 35 µL custom bead suspension, 90 µL AMPure XP bead suspension (Beckman Coulter) were used as above. Incubation on the Hula mixer was performed for 20 min. After washing twice with 70% (v/v) ethanol and subsequent drying, the pellet was resuspended in 20 µL of nuclease- free water and the DNA concentration was determined once again using a Qubit fluorometer. Integrity and fragment length distribution of the obtained wgaDNA samples were analysed with the 4150 TapeStation System using the Genomic DNA reagents and ScreenTape (Agilent Technologies).

Illumina Sequencing and computational analysis

Samples of “*Ca.* U. amorphum” and both amoebae obtained via WGA, as well as the DNA sample obtained from JCM (catalogue number: JGD18741) were sequenced at Eurofins Genomics with an Illumina NovaSeq Sequencer in NovaSeq 6000 S4 PE150 X mode. 30-35 million read pairs were obtained for each sample.

Obtained Illumina sequencing reads were subsequently trimmed with Trimmomatic v0.40 ^1^ and paired-end reads were merged with FLASH v.1.2.11 ^2^. Subsequent assembly of trimmed/merged reads was performed with MEGAHIT v1.2.9 ^3^. Consecutively, mappings were calculated with BamM v1.7.3 (<https://github.com/ecogenomics/BamM>) while binning was performed with MaxBin2 v2.2.7 ^4^ and MetaBAT 2 v2.12.1 ^5^. Results were classified with the classify_wf workflow of GTDB-Tk v1.6.0

Long-read sequencing with Oxford Nanopore and (meta-)genome assembly

For nanopore long-read sequencing, a multiplex sequencing library was prepared from 800 to 1000 ng of debranched wgaDNA according to the manufacturer’s protocol for the Native Barcoding Kit 24 (SQK-NBD114.24, Oxford Nanopore Technologies) with few modifications. Briefly, the DNA was repaired using the NEBNext FFPE DNA Repair Mix and the NEBNext Ultra II End Repair / dA-tailing Module reagents (New England Biolabs). The incubation times at 65 °C and 20 °C were extended to 20 min each and all incubation times on the Hula mixer during clean-up with AMPure XP Beads (Beckman Coulter) were prolonged to 10 min. NEB Blunt/TA Ligase Mix (New England Biolabs) was used for native barcode ligation, which was stopped after 30 min with EDTA. All samples were pooled and cleaned up using AMPure XP Beads (Beckman Coulter). Sequencing adapters were ligated for 30 min to the DNA using the NEBNext Quick Ligation Module (New England Biolabs) for 30 min, followed by another purification step with AMPure XP Beads (Beckman Coulter). Thereby, the washing steps were performed with Long Fragment Buffer (Oxford Nanopore Technologies) instead of 80% (v/v) ethanol to enrich DNA fragments larger than 3 kb. Sequencing was performed on a MinION Mk1B device (Oxford Nanopore Technologies) with a R10.4.1 flowcell (FLO-MIN114, Oxford Nanopore Technologies).

Raw nanopore sequencing data was basecalled using Dorado basecaller version 0.5.3 with basecalling model dna_r10.4.1_e8.2_400bps_sup@v4.3.0 and the additional flag “--min-qscore 10” for filtering reads with a Phred quality score ≥ 10 (Oxford Nanopore Technologies). Demultiplexing and adapter trimming were performed using Dorado demux version 0.5.3 (Oxford Nanopore Technologies). Reads were uploaded to the Galaxy web platform, and the server available under the public domain usegalaxy.eu was used for the processing of the data ^6^. We created and used a Galaxy workflow that included NanoPlot version 1.41.0 and FastQC version 0.74 for quality control of nanopore reads (https://www.bioinformatics.babraham.ac.uk/projects/fastqc/), Flye version 2.9.1 with the optional flags “--nano-hq”, “--scaffolds” and “--meta” ^7,8^ for (meta-) genome assembly and scaffolding. Medaka version 1.7.2 (Oxford Nanopore Technologies) with the model “r1041_e82_400bps_sup_g615” was used for polishing the raw assembly with the original long reads. Obtained contigs and scaffolds were binned using MetaBAT2 version 2.15 with a tabular depth matrix file listing mean and variance of the base coverage depth (optional flag “--abdFile”) ^5^. Completeness of the resulting bins was evaluated using BUSCO version 5.4.6 ^9^ with auto-detection of the lineage and the genome was annotated using Prokka version 1.14.6 ^10,11^. The BUSCO completeness of Bin02 (containing the contigs belonging to ”*Ca*. U. helgolandensis” Hl_Enr7) was compared to the BUSCO completeness of the reference genome of “*C.* U.amorphum” SRT547 (NCBI RefSeq assembly accession: GCF_009002475.1). The 16S rRNA gene sequences were analysed using the NCBI blastn suite against the nucleotide collection using Megablast ^12^. For Bin05, which did not contain a 16S rRNA gene sequence, the nucleotide sequence of the ribose-phosphate pyrophosphokinase gene was used for Megablast ^12^.

Analysis of phylogenetic markers and tree reconstruction

The 16S rRNA gene sequence of ”*Ca*. U. helgolandensis” Hl_Enr7 was extracted from the annotated genome annotated with NCBI’s PGAP pipeline and the identification of the closest neighbours was performed using NCBI BLAST ^13^. The 16S rRNA gene sequences of the novel isolate and all current members of the phylum *Planctomycetota* were aligned with ClustalW ^14^. The 16S rRNA gene sequence-based maximum likelihood phylogenetic tree was calculated from the alignment with FastTree 2.1 ^15^ employing the GTR+CAT model and 1000 bootstraps replications. Three 16S rRNA genes of bacterial strains from the PVC (*Planctomycetota-Verrucomicrobiota-Chlamydiota*) superphylum outside of the phylum *Planctomycetota*, namely *Opitutus terrae* (NCBI acc. no. AJ229235), *Kiritimatiella glycovorans* (acc. no. NR_146840) and *Lentisphaera araneosa* (acc. no. NR_027571), were used as outgroup. The multi-locus sequence analysis (MLSA)-based phylogenetic tree was constructed using autoMLST with 500 bootstrap replicates ^16^. The analysis was performed with the autoMLST-simplified-wrapper tool available on GitHub (https://github.com/KatSteinke/automlst-simplified-wrapper). Tree reconstruction included all reference genomes of strains belonging to the current phylum *Planctomycetota* and the NCBI reference genomes of *Opitutus terrae* (GenBank acc. no. GCA_000019965.1), *Kiritimatiella glycovorans* (GCA_001017655.1) and *Lentisphaera araneosa* (GCA_000170755.1) served as outgroup. Average amino acid identities (AAI) and average nucleotide identities (ANI) were calculated using the respective scripts of the enveomics collection ^17^. The percentage of conserved proteins (POCP) was calculated as described ^18^. The *rpoB* gene sequences were taken from the annotated genomes and sequence identities were determined as previously described ^19^. Alignment and matrix calculation were performed with Clustal Omega ^20^ upon extracting a ca. 1300 bp region of the *rpoB* coding sequence that would have been sequenced with the described primer set. The *in silico* DNA-DNA hybridisation (DDH) value was calculated using the Genome-to-Genome Distance Calculator ^21^.

Genome annotation and pangenome analysis

The obtained genome of the novel isolate was annotated using the most recent version of the NCBI PGAP pipeline (version 2024-04-27.build7426). Coding density and DNA G+C content were analysed with CheckM v1.1.6 ^22^. The other genomic features (numbers of protein-coding gene and rRNAs, etc.) were obtained from the PGAP-annotated genomes. The pangenome analysis of the novel isolate and “*Ca*. U. amorphum” was performed with anvi’o v. 8 ^23^

Kegg KOfam analysis

Genomes of seven species employing FtsZ-based cell division (*Bacillus subtilis* 168, *Streptococcus pneumoniae* Hu17, *Myxococcus xanthus* DK_1622, *Escherichia coli* O157H7, *Caulobacter crescentus* NA1000, *Hyphomonas neptunia* ATCC_15444, *Streptomyces coelicolor* ATCC 23899) and one species per order of the phylum *Planctomycetota* (*Stieleria maiorica* Mal15, *Planctopirus limnophila* DSM_3776, “*Kueselia aquiterrae*” EP7, *Gemmata obscuriglobus* UQM_2246, *Mucisphaera calidilacus* Pan265, *Sedimentisphaera salicampi* ST-PulAB-D4, *Humisphaera borealis* M1803, “*Engelhardtia mirabilis”* Pla133, “Ca. *Kueninia stuttgartensis*” CSTR1, “*Ca*. U. amorphum” SRT547) as well as “*Rhohdeia mirabilis”* Pla163 and “*Saltatorellus ferox”* Poly30 were obtained from the NCBI database. KEGG KOfams were obtained with the “Estimate Metabolism” function of anvi’o v. 7.1 ^23^. The presence of genes was analysed via the presence of the respective KEGG KOfam numbers in the anvi’o output (*mraZ*/K03925, *mraW*/K03438, *ftsL*/K03586, *ftsI*/K03587, *murE*/K01928, *murF*/K01929, *mraY*/K01000, *murD*/K01925, *ftsW*/K03588, *murG*/K02563, *murC*/K01924, *ddl*/K01921, *ftsQ*/K03589, *ftsA*/K03590, *ftsZ*/K03531, *mreB*/K03569, *mreC*/K03570, *mreD*/K03571, *rodA*/K05837, *ftsK*/K03466, *rodZ*/K15539, *murJ*/K03980, *murA*/K00075, *murB*/K00790 and *alr*/K01755).

Supplementary Results and Discussion

Feeding Experiments

*“Ca.* U. amorphum” was mainly incubated with *A. macleodii* as prey bacterium. However, we observed internalisation of other bacteria such as *E. coli*, and the planctomycetal species *Stieleria maiorica* Mal15^T^ as well. In enrichment cultures, internalisation of various morphological distinct bacteria by strain HlEnr_7 was observed. Neither dead *A. macleodii* cells, killed by UV radiation or heat inactivation (65°C, 30 min), nor an extract of *A. macleodii* cells did support growth of “*Ca.* U. amorphum”.

**Description of ”*Candidatus* Uabimicrobium helgolandensis” sp. nov.**

”*Candidatus* Uabimicrobium helgolandensis” (hel.go.lan.den’sis. N.L. fem. adj. *helgolandensis*, of Heligoland, corresponding to the origin of the strain from the German island Heligoland).

Cells have a size of 4-20 µm and maintain a mostly round cell shape during crawling. They require a surface to generate the force needed for dividing into two daughter cells. Opposite cell poles move apart until they are only connected by a thin, thread-like structure. Biomass formation is dependent on the internalisation of living bacteria. Dead bacterial biomass or bacterial extracts did not support growth. The type strain is HlEnr_7, which was isolated from surface water of the North Sea sampled in the harbor of Heligoland Island. The type strain has a genome size of 9.32 Mb and a DNA G+C content of 34.9%.

Supplementary Tables

**Supplementary Table 1.** Overview of the analysed phylogenetic markers.

| Marker | Value (%)^1^ | Threshold |
| --- | --- | --- |
| 16S rRNA gene | 97.0 | same species: >98.7 %^24^  same genus: >94.5 %^24^ |
| ANI | 77.6 | same species: >95 %^25^ |
| AAI | 71.6 | same species: >85 %^26^  same genus: 60-80 %^26^ |
| POCP | 69.9 | same genus: >50 %^18^ |
| DDH | 14.9 | same species: >70 % ^17^ |
| *rpoB* | 82.8 | same species: >96.3 % ^27^  same genus: >75.5-78.0 %^27^ |

^1^for comparison of *Ca.*U. amorphum SRT547 and “*Ca.* U. helgolandensis” HlEnr_7

**Supplementary Table 2.** Comparison of genomic characteristics of “*Ca*. U. helgolandensis” HlEnr_7 and “*Ca*. U. amorphum” SRT547.

| Feature | HlEnr_7 | SRT547 |
| --- | --- | --- |
| Genome size (bp) | 9,323,131 | 9,503,417 |
| Number of contigs | 1 | 1 |
| DNA G+C content (%) | 34.9 | 39.4 |
| Coding density (%) | 87.5 | 91.4 |
| Genes (total) | 6,846 | 6,688 |
| Protein-coding genes | 6,659 | 6,582 |
| Hypothetical proteins | 2,056 | 2,916 |
| Hypothetical proteins (%) | 30.8 | 44.3 |
| rRNA genes (5S-16S-23S) | 4-4-4 | 3-3-3 |
| tRNAs | 86 | 76 |
| CRISPR arrays | 1 | 1 |
| BUSCO completeness with the lineage dataset bacteria_odb10 (%) | 79.8 | 81.4 |

Supplementary Figures

**Supplementary Figure 1.** Cell division of *Ca.* U. amorphum, *Hartmannella sp.* CCAP 1534/15 and *S. japonica* CCAP 1593/1. Cells of *Ca.* U. amorphum (A) continue to walk apart until newly emerging daughter cells are only connected by a thin, thread-like structure (red arrows) that eventually tears. *Hartmannella sp.* CCAP 1534/15 (B) elongates as well, however, the poles stop moving apart before constriction at mid-cell starts. The remaining connection (red arrows) becomes thinner as observed for *Ca.* U. amorphum but is severed without applying force of the two daughter cells walking apart. In case of *S. japonica* CCAP1593/1 (C) no elongation seems to be necessary, and cells constrict from two sides at mid-cell (redarrows). Full separation of the emerging daughter cells is achieved without any spatial distancing during the process. Scale bars are 2 μm (A) and 5 μm (B, C). Timestamps are in hh:mm:ss format.


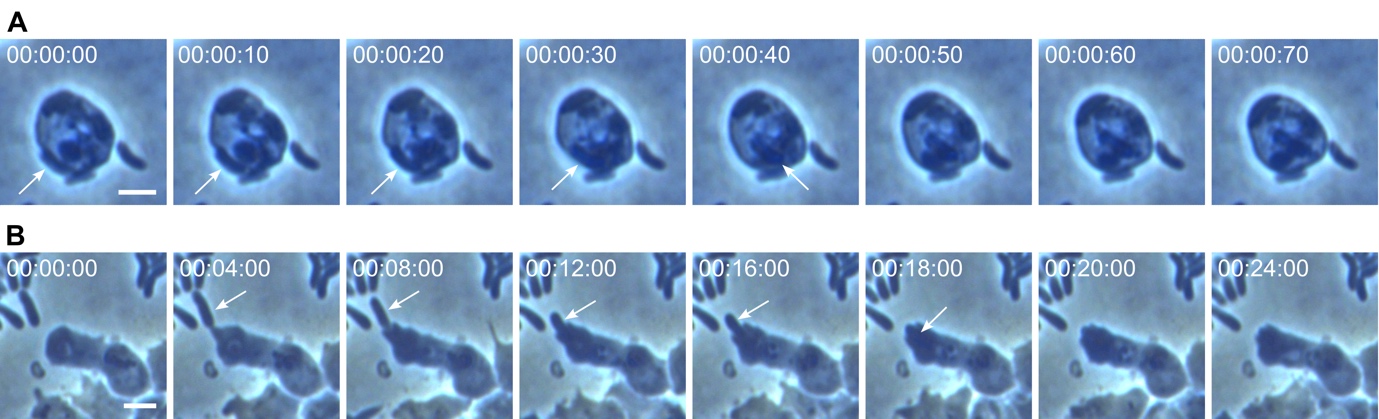


**Supplementary** **Figure 2**. Prey uptake of *Ca.* U. amorphum (A) and *S. japonica* CCAP 1593/1 (B). Prey bacteria engulfed by the respective predator are indicated with white arrows. Scale bars are 2 μm. Timestamps are in hh:mm:ss format.


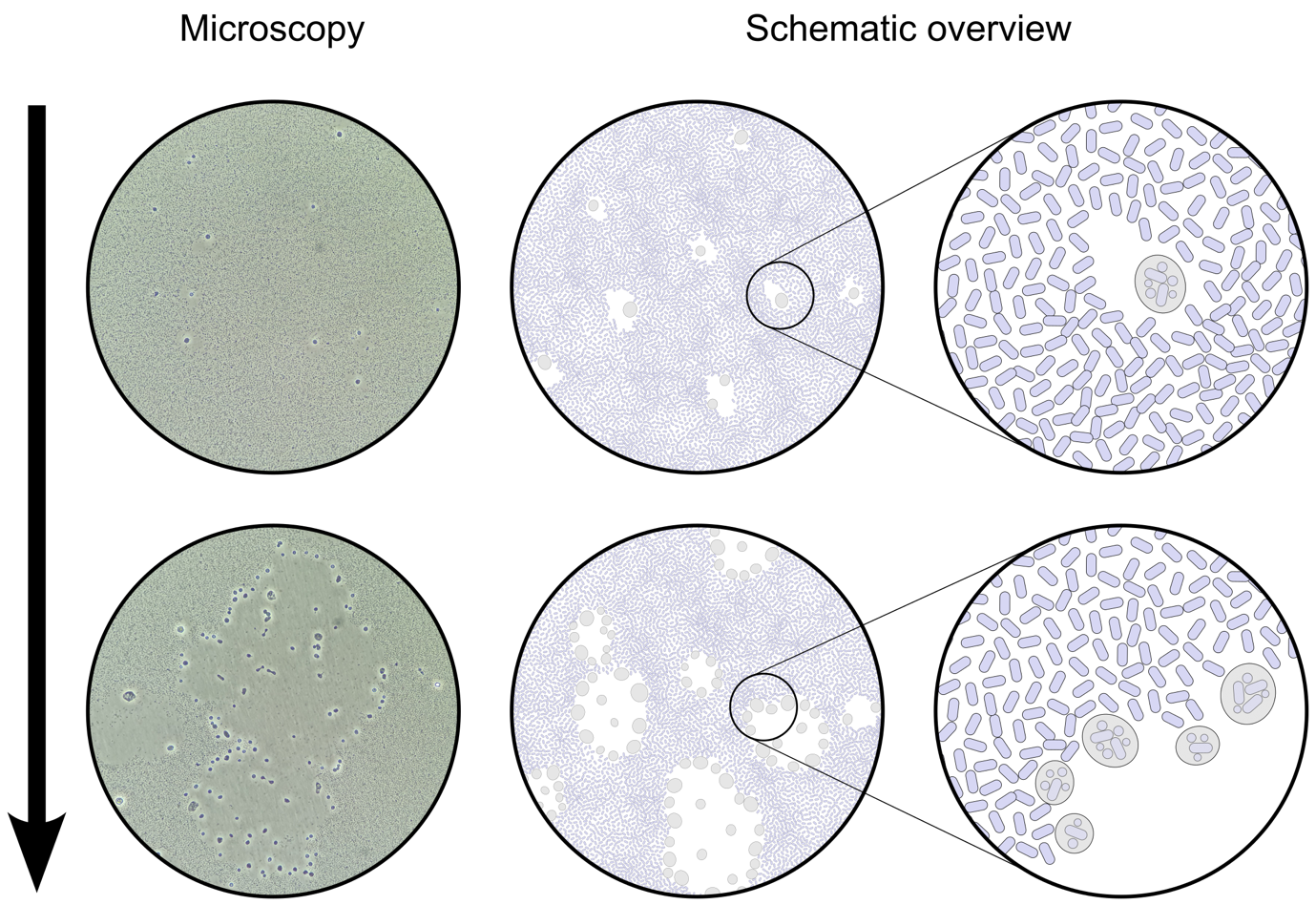


**Supplementary Figure 3.** “Feeding circles” of *Ca.* U. amorphum SRT547. Cells (microscopy: larger dark spots, schematic: grey) feed only in their close proximity but very efficiently, causing spots without any bacteria (plaques). With increasing cell number, the size of the plaques increases and cells are mostly feeding at the rim, thereby forming “feeding circles”. Only few cells remain in the middle of such plaques.


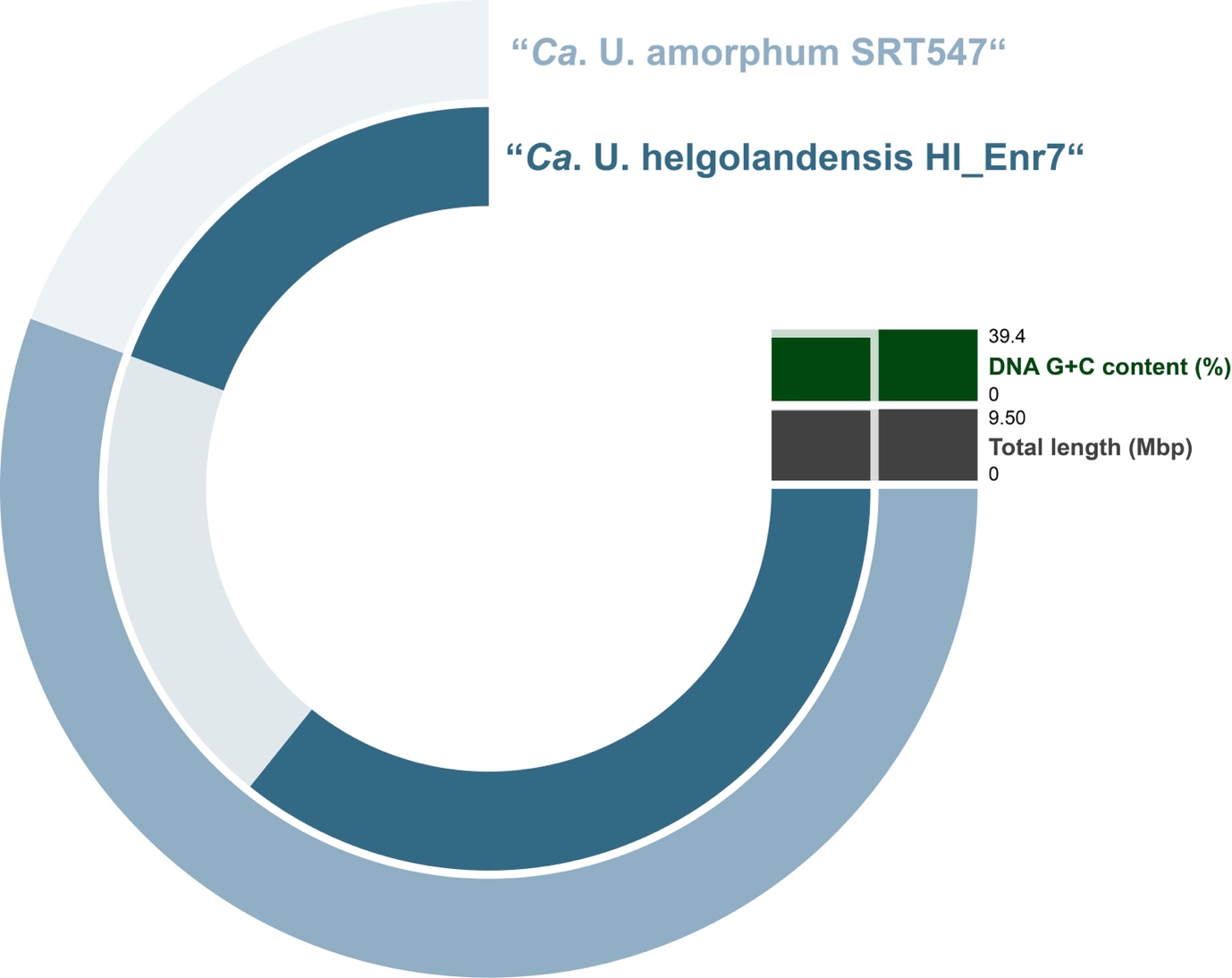


**Supplementary Figure 4.** Pangenome of “*Ca*. U. helgolandensis” HlEnr_7 and *Ca*.U. amorphum SRT547.

**
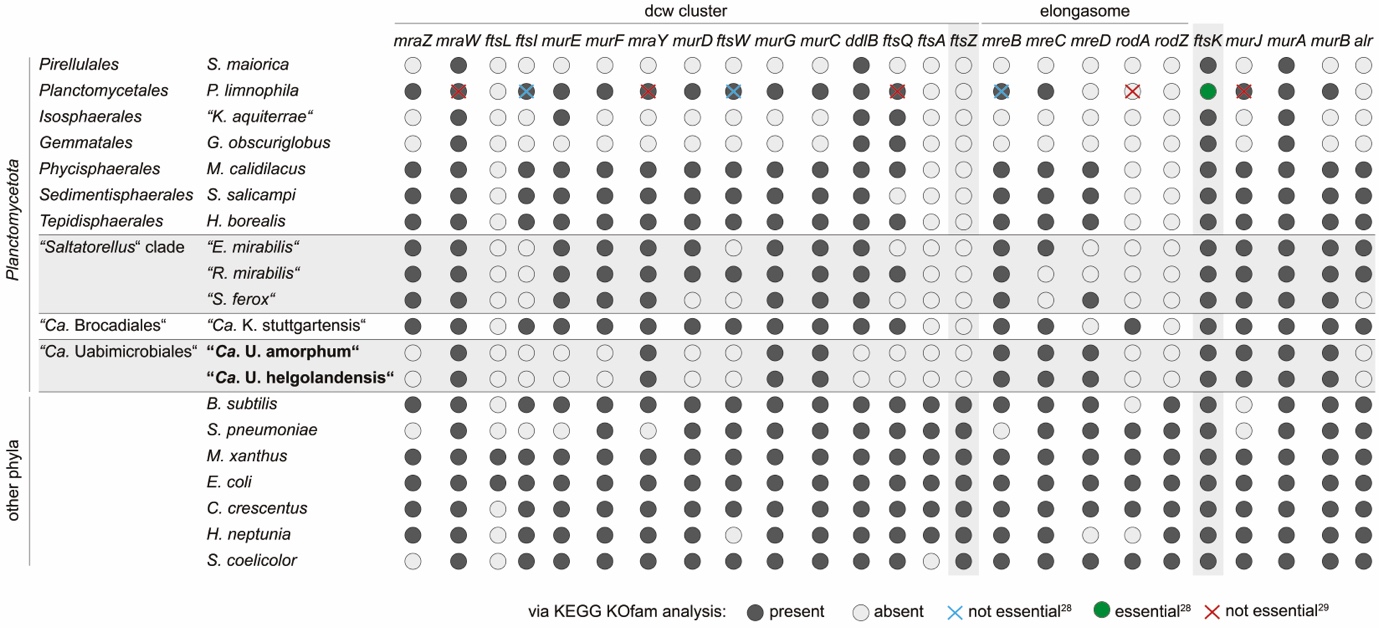
**

**Supplementary Figure 5.** KEGG KOfam analysis of canonical cell division and peptidoglycan synthesis genes (including the dcw cluster and the elongasome) of “*Ca*. U. amorphum” and “*Ca*. U. helgolandensis”. One strain for each order of the phylum *Planctomycetota* and seven species employing FtsZ-based division were compared. In contrast to the species employing FtsZ-based cell division, in members of the phylum *Planctomycetota* key genes of the dcw cluster seem to be missing (*ftsL*, *ftsZ*, *ftsA*) or can be deleted without affecting morphology (*mraW*, *mraY*, *ftsQ*, *rodA*, *murJ*) ^28^. The gene *ftsK* encoding for a DNA translocase cannot be deleted and thus appears to be essential^29^.

Supplementary Movies

**Supplementary Movie 1**. Endocytosis-like prey uptake of “*Ca.* U. amorphum” (large cells). Prey uptake takes place in the following order: lower cell, left upper cell, lower cell.

**Supplementary Movie 2**. Cell division of “*Ca.* U. amorphum”. After moving around, the cell settles and starts to extend in two opposite directions. The newly developing daughter cells move apart until only a thin, thread-like connection remains, which eventually disrupts.

**Supplementary Movie 3**. Cell division of *Hartmannella* sp. CCAP 1534/15. After moving around, the cell settles, where it remains for a while in a rather round shape. Finally, the cell elongates by expanding into two opposite directions and forms two separate daughter cells. However, the distance between the developing daughter cells is rather low in comparison to “*Ca.* U. amorphum” and no thread-like connection is developed.

**Supplementary Movie 4**. Phagocytosis of *Squamamoeba japonica* CCAP 1493/1. The elongated cell in the middle of the image extends towards the bacterium laying closest to its tip and takes it up via phagocytosis.

Literature

1. Bolger, A. M., Lohse, M. & Usadel, B. Trimmomatic: a flexible trimmer for Illumina sequence data. *Bioinformatics* **30**, 2114-2120 (2014). <https://doi.org:10.1093/bioinformatics/btu170>
2. Magoč, T. & Salzberg, S. L. FLASH: fast length adjustment of short reads to improve genome assemblies. *Bioinformatics* **27**, 2957-2963 (2011). <https://doi.org:10.1093/bioinformatics/btr507>
3. Li, D., Liu, C.-M., Luo, R., Sadakane, K. & Lam, T.-W. MEGAHIT: an ultra-fast single-node solution for large and complex metagenomics assembly via succinct de Bruijn graph. *Bioinformatics* **31**, 1674-1676 (2015). <https://doi.org:10.1093/bioinformatics/btv033>
4. Wu, Y.-W., Simmons, B. A. & Singer, S. W. MaxBin 2.0: an automated binning algorithm to recover genomes from multiple metagenomic datasets. *Bioinformatics* **32**, 605-607 (2015). <https://doi.org:10.1093/bioinformatics/btv638>
5. Kang, D. D. *et al.* MetaBAT 2: an adaptive binning algorithm for robust and efficient genome reconstruction from metagenome assemblies. *PeerJ* **7**, e7359 (2019). <https://doi.org:10.7717/peerj.7359>
6. The Galaxy Community. The Galaxy platform for accessible, reproducible and collaborative biomedical analyses: 2022 update. *Nucleic Acids Res.* **50**, W345-W351 (2022). <https://doi.org:10.1093/nar/gkac247>
7. Lin, Y. *et al.* Assembly of long error-prone reads using de Bruijn graphs. *Proceedings of the National Academy of Sciences* **113**, E8396-E8405 (2016). <https://doi.org:doi:10.1073/pnas.1604560113>
8. Kolmogorov, M., Yuan, J., Lin, Y. & Pevzner, P. A. Assembly of long, error-prone reads using repeat graphs. *Nat. Biotechnol.* **37**, 540-546 (2019). <https://doi.org:10.1038/s41587-019-0072-8>
9. Simão, F. A., Waterhouse, R. M., Ioannidis, P., Kriventseva, E. V. & Zdobnov, E. M. BUSCO: assessing genome assembly and annotation completeness with single-copy orthologs. *Bioinformatics* **31**, 3210-3212 (2015). <https://doi.org:10.1093/bioinformatics/btv351>

10 Cuccuru, G. *et al.* Orione, a web-based framework for NGS analysis in microbiology. *Bioinformatics* **30**, 1928-1929 (2014). https://doi.org:10.1093/bioinformatics/btu135

11 Seemann, T. Prokka: rapid prokaryotic genome annotation. *Bioinformatics* **30**, 2068-2069 (2014). https://doi.org:10.1093/bioinformatics/btu153

12 Zhang, Z., Schwartz, S., Wagner, L. & Miller, W. A Greedy Algorithm for Aligning DNA

Sequences. *J. Comput. Biol.* **7**, 203-214 (2000). <https://doi.org:10.1089/10665270050081478>

13 Johnson, M. *et al.* NCBI BLAST: a better web interface. *Nucleic Acids Research* **36**, W5-W9 (2008). <https://doi.org:10.1093/nar/gkn201>

14 Thompson, J. D., Gibson, T. J. & Higgins, D. G. Multiple Sequence Alignment Using ClustalW and ClustalX. *Current Protocols in Bioinformatics* **00**, 2.3.1-2.3.22 (2003). <https://doi.org:https://doi.org/10.1002/0471250953.bi0203s00>

15 Price, M. N., Dehal, P. S. & Arkin, A. P. FastTree 2 – Approximately Maximum-Likelihood Trees for Large Alignments. *PLOS ONE* **5**, e9490 (2010). <https://doi.org:10.1371/journal.pone.0009490>

16 Alanjary, M., Steinke, K. & Ziemert, N. AutoMLST: an automated web server for generating multi-locus species trees highlighting natural product potential. *Nucleic Acids Res.* **47**, W276-W282 (2019). <https://doi.org:10.1093/nar/gkz282>

17 Rodriguez-R, L. M. & Konstantinidis, K. T. The enveomics collection: a toolbox for specialized analyses of microbial genomes and metagenomes. Report No. 2167-9843, (PeerJ Preprints, 2016).

18 Qin, Q.-L. *et al.* A proposed genus boundary for the prokaryotes based on genomic insights. *J. Bacteriol.* **196**, 2210-2215 (2014). <https://doi.org:10.1128/JB.01688-14>

19 Bondoso, J., Harder, J. & Lage, O. M. rpoB gene as a novel molecular marker to infer phylogeny in Planctomycetales. *Antonie van Leeuwenhoek* **104**, 477-488 (2013). <https://doi.org:10.1007/s10482-013-9980-7>

20 Sievers, F. & Higgins, D. G. Clustal Omega. *Current Protocols in Bioinformatics* **48**, 3.13.11-13.13.16 (2014). <https://doi.org:https://doi.org/10.1002/0471250953.bi0313s48>

21 Meier-Kolthoff, J. P., Carbasse, J. S., Peinado-Olarte, R. L. & Göker, M. TYGS and LPSN: a database tandem for fast and reliable genome-based classification and nomenclature of prokaryotes. *Nucleic Acids Res.* **50**, D801-D807 (2021). <https://doi.org:10.1093/nar/gkab902>

22 Parks, D. H., Imelfort, M., Skennerton, C. T., Hugenholtz, P. & Tyson, G. W. CheckM: assessing the quality of microbial genomes recovered from isolates, single cells, and metagenomes. *Genome Research* **25**, 1043-1055 (2015). <https://doi.org:10.1101/gr.186072.114>

23 Eren, A. M. *et al.* Community-led, integrated, reproducible multi-omics with anvi’o. *Nature Microbiology* **6**, 3-6 (2021). <https://doi.org:10.1038/s41564-020-00834-3>

24 Yarza, P. *et al.* Uniting the classification of cultured and uncultured bacteria and archaea using 16S rRNA gene sequences. *Nat. Rev. Microbiol.* **12**, 635-645 (2014). <https://doi.org:10.1038/nrmicro3330>

25 Rodríguez-R, L. & Konstantinidis, K. Bypassing Cultivation To Identify Bacterial Species: Culture-independent genomic approaches identify credibly distinct clusters, avoid cultivation bias, and provide true insights into microbial species. *Microbe Magazine* **9**, 111-118 (2014). <https://doi.org:10.1128/microbe.9.111.1>

26 Luo, C., Rodriguez-R, L. M. & Konstantinidis, K. T. MyTaxa: an advanced taxonomic classifier for genomic and metagenomic sequences. *Nucleic Acids Res.* **42**, e73-e73 (2014). <https://doi.org:10.1093/nar/gku169>

27 Kallscheuer, N. *et al.* Description of three bacterial strains belonging to the new genus *Novipirellula* gen. nov., reclassificiation of *Rhodopirellula rosea* and *Rhodopirellula caenicola* and readjustment of the genus threshold of the phylogenetic marker *rpoB* for *Planctomycetaceae*. *Antonie Van Leeuwenhoek* **113**, 1779-1795 (2020). <https://doi.org:10.1007/s10482-019-01374-5>

28 Rivas-Marin, E., Moyano-Palazuelo, D., Henriques, V., Merino, E. & Devos, D. P. Essential gene complement of Planctopirus limnophila from the bacterial phylum Planctomycetes. *Nature Communications* **14**, 7224 (2023). <https://doi.org:10.1038/s41467-023-43096-3>

29 Rivas-Marin, E. *et al.* Non-essentiality of canonical cell division genes in the planctomycete Planctopirus limnophila. *Scientific Reports* **10** (2020). https://doi.org:10.1038/s41598-019-56978-8
